# Commentary: There's more than one way to skin a cat (thoraco)

**DOI:** 10.1016/j.xjtc.2021.03.025

**Published:** 2021-03-26

**Authors:** Marvin D. Atkins, Michael J. Reardon

**Affiliations:** Department of Cardiovascular Surgery, Houston Methodist Hospital, Houston, Tex

**Figure:**
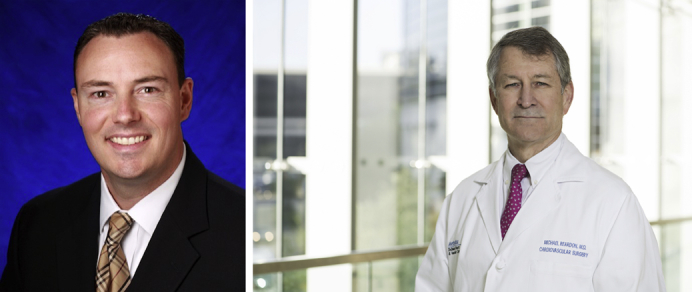
Marvin D. Atkins, MD, and Michael J. Reardon, MD

**Central Message**

Thoracoabdominal aortic aneurysm repair is a complex procedure that has a number of different technical approaches to achieve success. Avoiding ischemia in all areas is central to all.

See Article page 29.

Open thoracoabdominal aortic aneurysm repair has been one of the most demanding operations performed, both for the surgeon and the patient, since its original description by Etheridge and colleagues in 1955^1^ and popularized at our institution by Crawford and colleagues in the 1960s.^2^ Over the years, multiple variations in the steps of the operation and various adjuncts have been proposed to improve on the results and minimize the complications associated with these complex procedures. Perfusion adjuncts to avoid cardiopulmonary bypass and pump dose systemic heparinization have included the Gott shunt,^3^ in-line mesenteric shunting as described by Cambria and colleagues,^4^ and partial left heart bypass. The technique of “clamp and sew” for Crawford extent 1, 2, and 3 thoracoabdominal aortic aneurysm has for the most part been abandoned owing to the increased risk of spinal cord ischemia and visceral malperfusion.

In the current era of open thoracoabdominal aortic aneurysm repair, widely performed treatment perfusion strategies include full cardiopulmonary bypass, with or without deep hypothermia, and partial left heart bypass with sequential clamping. In this issue of *JTCVS Techniques*, Hiremath and colleagues^5^ describe the modified branch first technique of sequential mesenteric and left renal artery debranching with perfusion, followed by proximal and then distal aortic anastomoses. The technique involves cannulation of the descending thoracic aorta above the aneurysm or using left heart bypass with placement of the outflow cannula in the right renal limb of a branched thoracoabdominal graft. The celiac, superior mesenteric artery, and left renal artery are then sequentially debranched and perfused through the cannulated graft. This technique requires dissection of the mesenteric vessels and left renal artery well beyond their origins to place proximal and distal clamps and enough artery in between to sew to. In some patients, distal dissection of the celiac and superior mesenteric artery may prove quite difficult.

The authors propose that this technique limits spinal cord ischemia, as the aorta is perfused with pulsatile blood throughout the debranching portion of the procedure. The perfusion is then stopped before completion of the right renal artery bypass. The cannula is removed from the graft, and that limb is used for the right renal bypass. This portion of the procedure is similar to the technique described by Coselli,^6^ in which partial left heart bypass is transitioned to selective visceral perfusion via ostial cannulas and then stopped before the distal anastomosis. Our preferred technique is to continue perfusion to the distal aorta and iliac arteries throughout the operation, typically via left femoral artery cannulation. Griepp and Griepp^7^ have contributed extensively to our understanding of spinal cord perfusion, the “collateral network concept,” and the importance of vertebral and iliac artery contributions to spinal cord perfusion.

Although the technique the authors describe is novel and avoids cardiopulmonary bypass, no definitive conclusions can be made about the purported benefits of spinal cord ischemia and visceral malperfusion. Clearly, when it comes to open thoracoabdominal aortic aneurysm repair, there is more than one way to skin a cat (thoraco).
